# A multiplexed, systems-based approach for prediction of antibody neutralization breadth for soluble human receptors

**DOI:** 10.1093/jimmun/vkag106

**Published:** 2026-05-25

**Authors:** Qixin Wang, Lindsay R McManus, Kate S Levine, Ross Blanc, Hadar Malca, Brian A Joughin, Caihong Bi, Keerti, Douglas A Lauffenburger, Duane R Wesemann, Ryan P McNamara

**Affiliations:** Department of Immunology and Infectious Diseases, Harvard T.H. Chan School of Public Health, Boston, MA, United States; Department of Immunology and Infectious Diseases, Harvard T.H. Chan School of Public Health, Boston, MA, United States; Department of Immunology and Infectious Diseases, Harvard T.H. Chan School of Public Health, Boston, MA, United States; Department of Immunology and Infectious Diseases, Harvard T.H. Chan School of Public Health, Boston, MA, United States; Department of Immunology and Infectious Diseases, Harvard T.H. Chan School of Public Health, Boston, MA, United States; Department of Biological Engineering, Massachusetts Institute of Technology, Cambridge, MA, United States; Division of Allergy and Immunology, Division of Genetics, Department of Medicine, Brigham and Women’s Hospital, Harvard Medical School, Boston, MA, United States; Division of Allergy and Immunology, Division of Genetics, Department of Medicine, Brigham and Women’s Hospital, Harvard Medical School, Boston, MA, United States; Department of Biological Engineering, Massachusetts Institute of Technology, Cambridge, MA, United States; Division of Allergy and Immunology, Division of Genetics, Department of Medicine, Brigham and Women’s Hospital, Harvard Medical School, Boston, MA, United States; Ragon Institute of Mass General, MIT, and Harvard, Cambridge, MA, United States; Department of Immunology and Infectious Diseases, Harvard T.H. Chan School of Public Health, Boston, MA, United States

**Keywords:** correlates of protection, Fc receptor, neutralization, prediction, systems serology

## Abstract

For endemically circulating viruses, quantifying neutralization capacity of antibodies in a rapid and high-throughput manner is paramount given the ever-evolving targets of neutralization. Moreover, identifying features of an antibody response correlating with neutralization at the multivariate level can inform vaccine boosting strategies and next-generation monoclonal antibody therapies. In this study, we developed a systems-predicted neutralizing antibody platform that is capable of quantifying neutralization capacity at the multiplex level. We validated the platform using SARS-CoV-2 ancestral and highly diverged Omicron sublineage spikes simultaneously. Combining these results with a broader systems serology approach identified humoral signatures that predict broadly neutralizing responses that are divergent between vaccine or hybrid immunity. In both cases, though, predicted neutralization responses were enmeshed in antibody networks, showing that target-specific and broadly neutralizing antibody responses are multi-isotype- and subclass-influenced. We propose that neutralization against a diverse array of viral receptor-binding proteins can be quantified using a systems-based platform in a sample- and time-sparing approach, which can be further employed to predict protection against newly emerged viruses or variants.

## Graphical abstract

**Graphical Abstract:**
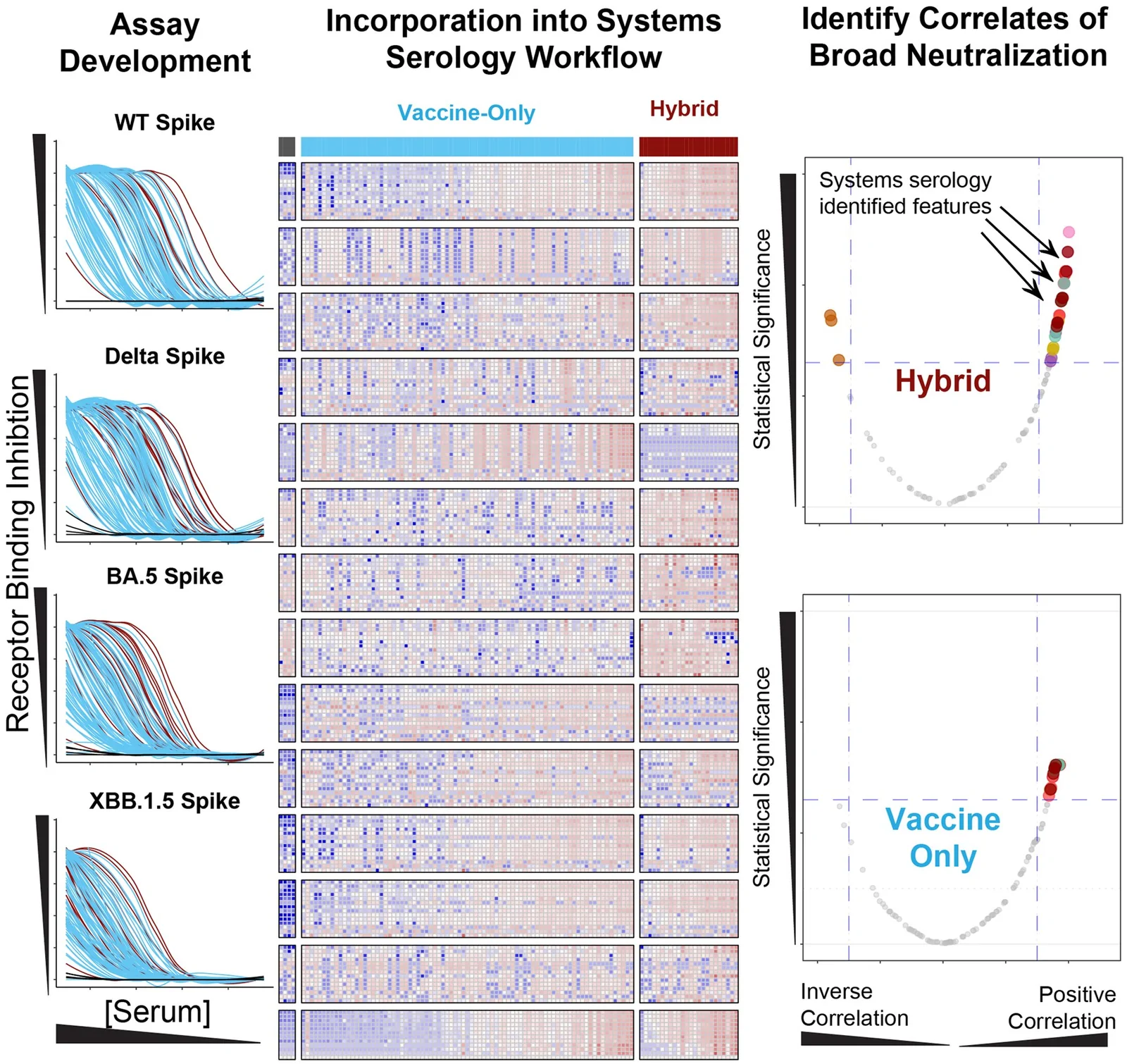

## Introduction

Antibodies are frontline immune defenders to respiratory pathogens, exerting many protective roles, including direct neutralization, effector-based functions, complement deposition, and others.^1–6^ As such, vaccinations against respiratory viruses have sought to leverage antibody polyfunctionality to confer protection. Many vaccination strategies have focused on bolstering neutralizing antibody responses by presenting the virus receptor-binding proteins in a pre-fusion state, with neutralization targets/epitopes more exposed.^7–12^

The rapid development and deployment of the coronavirus disease 2019 (COVID-19) vaccines is one of the crowning achievements of medical innovation of the 21st century. Rational antigen presentation coupled with innovative delivery platforms afforded high levels of protection against COVID-19, particularly lower respiratory tract infection.^8^^,^^13–15^ Importantly, those who were infected with severe acute respiratory syndrome coronavirus 2 (SARS-CoV-2) and recovered could be boosted with a vaccination, conferring a “hybrid” immunity that exhibited strong protection.^16–20^

The presence of neutralizing antibodies has been identified as a correlate of protection against respiratory illnesses.^21–25^ As SARS-CoV-2 adapted to its new human host, many mutations accumulated in the spike protein that evaded antibody neutralization and enhanced the virus’s ability to penetrate respiratory endothelial cells and to undergo human-to-human transmission.^26–36^ To that end, vaccine boosters were developed and employed to expand immune recognition of antigenically drifted variants.^31^^,^^33^^,^^37–42^ The predicted effectiveness of these boosters against SARS-CoV-2, and boosters against other respiratory viruses, continues to be largely quantified through neutralization-based readouts. It should be acknowledged that other protective immune features against respiratory viruses have been identified, such as T cell–based recognition and antibody effector functions.^43–47^ Given the continuous drifting of neutralization targets, rapid and high-throughput assays that can quantify neutralization-based responses are needed to accelerate vaccine development and predict protection.

To address this gap, we developed an approach to rapidly quantify predicted neutralizing antibody levels using soluble human receptors. We validated this approach using spike trimers from SARS-CoV-2 variants simultaneously.^48^ This systems-based approach can be incorporated into a larger systems serology framework to identify features that correlated with neutralization capacities to individual variants, and determine what antibody signatures are correlated with broadly neutralizing responses. We found that vaccine- and hybrid-immune profiles have distinct broadly neutralizing signatures. Through this approach, we can quantify the neutralization capacities at the multiplex level in a time- and sample-sparing way, and predict broadly neutralizing signatures.

## Materials and methods

### Cohorts

Serum from human participants that was used in this study was previously described in Chen et al., 2022.^48^ Secondary use approval of the specimens was obtained by Harvard University. In brief, “hybrid immunity” was defined as COVID-19 convalescents who have been confirmed with SARS-CoV-2 infection by RT-PCR and received the mRNA vaccinations, and “vaccine-only immunity” was defined as COVID-19–naïve individuals who had received mRNA vaccination; blood was drawn multiple times after the first dose or different days after the second and third dose. A total of 4 pre-COVID-19 sera were included as negative serum samples. Further information on the cohort characteristics can be found in Table 1. The primary analysis endpoint was to quantify our systems-predicted neutralizing antibody (SNAb) approach with reported 50% neutralization titer (NT_50_) values independent of time since antigen exposure.

**Table 1.** Cohort overview of immune grouping.

Characteristic	Immune type	
	Vaccine immune	Hybrid immune
**Age, y**	42.7 (range, 24–75)	46.4 (range, 23–77)
**% male**	41	25
**% female**	59	75

#### System-neutralization antibody and serology assay

##### Antibody isotype and subclass binding profiling

Human antibody isotypes (IgG, IgM, IgA) and subclasses (IgG1, IgG2, IgG3, IgG4, IgA1, IgA2), along with Fc receptor (FcR) binding antibodies (FcγR2A, FcγR2B, FcγR3A, FcγR3B, and FcαR), were quantified for binding to the targeted antigens (Table S1). Antibody binding levels were determined as previously described.^66–69^ In brief, antigens were immobilized onto specific magnetic Luminex bead regions through N-hydroxysuccinimide esters (NHS esters) linkages (Thermo Fisher). The antigen-coupled microspheres were then incubated with appropriate concentrations of serum diluted in 1× phosphate-buffered saline (PBS), pH = 7.4, for 16 hours at 4 °C with continuous shaking in a sealed 384-well low-bind plate (Greiner). All incubations were done using 1X Assay buffer (1× PBS, 0.1% BSA, 0.02% Tween-20). Final dilutions of the antibody feature were determined through serial dilution curves to establish a linear range of detection. Plates were washed 3 times in 1X Assay buffer, and detection reagent (1:100 dilution of PE-coupled antibody in 1X Assay buffer) was added to the plate. The detection step was allowed to occur at room temperature for 1 hour with continuous shaking. Plates were then washed 3 times in 1X Assay buffer and then analyzed on the Luminex Intelliflex instrument (Diasorin).

Human FcRs were obtained from Duke University and biotinylated using the BirA500 kit (Avidity). Biotinylated receptors were purified using Zeba Spin columns (Thermo Fisher). To detect FcR-binding antibodies, the biotinylated receptors were added at the detection step along with streptavidin-PE. The detection step was allowed to proceed at room temperature for 1 hour with continuous shaking on the 384-well plate. After the incubation, the plates were washed 3 times with 1X Assay buffer and run on the Luminex Intelliflex instrument (Diasorin).

All samples were run in technical duplicates, and means were quantified and reported as median fluorescence intensity (MFI) in arbitrary units (AU). All data were subjected to quality controls. PBS wells (no serum added) were used to establish a lower limit of the blank. Background subtraction was performed on all analytes. As an additional level of quality control, technical replicates whose standard deviation was greater than 50% of the mean were not used for subsequent analysis. All quality control trimming was done using the systems processor for integration of data for research (SPIDR) version 1.0.

##### SNAb titer assay

Human angiotensin-converting enzyme 2 (ACE2) was biotinylated and incubated with spike-coated magnetic Luminex microspheres in increasing concentrations. Detection of the receptor:receptor-binding protein (spike:ACE2) was done similarly to above. A concentration of ACE2 that was within the linear range of detection for all spikes was used for modeling the binding inhibition. For specificity controls, beads were coated with human cytomegalovirus glycoprotein b (HCMV Gb), human influenza virus H1N1/Wisconsin/2022 (hemagglutinin [HA]), and Ebolavirus glycoprotein (Ebola GP). These viral antigens were used as binding specificity controls for ACE2. None of the specificity control antigens showed higher than baseline binding to ACE2 at the concentration used. Last, human CD4 was used as a receptor specificity control (Fig. S1).

Binding inhibition was modeled by incubating spike-coated microspheres with serially diluted serum samples from the cohorts, beginning at a 1:30 dilution. Serum samples were serially diluted in 3-fold dilutions length-wise along a 96-well plate. The diluted serum samples were added to the spike-containing microspheres in a 384-well plate and allowed to bind at room temperature for 1 hour with continuous shaking. Plates were washed similarly to our antibody binding assays. Detection with biotinylated and streptavidin-PE–coupled ACE2 was done at room temperature for 1 hour with continuous shaking in the 384-well plate. The plates were then washed, and binding was quantified on the Luminex Intelliflex instrument.

The SNAb titers were fitted into a 4-parameter logistic regression equation. The equation was required to achieve an *R*^2^ value >0.75. If a model was not achieved by this threshold, then the SNAb titer was reported as nonquantifiable. Each sample was then quantified for the dilution at which 50% binding inhibition to the spike occurred (SNAb titer) using the fitted curve. The binding inhibition was calculated as follows:


$$\mathrm{Binding}\;\mathrm{inhibition}\;\%=100\%\times (1-\frac{{\mathrm{Binding}}_{\mathrm{MFI}}-{\mathrm{Baseline}}_{\mathrm{MFI}}}{{\mathrm{Top}\;\mathrm{limit}}_{\mathrm{MFI}}-{\mathrm{Baseline}}_{\mathrm{MFI}}}).$$

The top limit MFI was defined as the mean signal from no-antibody (uninhibited binding) minus 3 standard deviations. Any detected signal (binding MFI) above this limit was normalized to the top limit MFI. The baseline MFI was the mean of the background control, or only the antigen-conjugated microspheres. Binding inhibition exceeding 100% was normalized to 100%. The reciprocal serum dilution at which binding inhibition was at 50% was used as a reported SNAb titer. All calculated SNAb titers that were lower than the most concentrated sample dilution (1:30) or nonquantifiable titers were reported as 30. Thus, a SNAb titer of 30 was a lower limit of quantitation. All SNAb titers were performed in technical replicates, similar to our antibody binding assay. For grouped analysis, median and individual titers were reported.

Comparisons between our SNAb assay and previously reported NT_50_ values^48^ were done using a Pearson correlation coefficient. *R* values of the correlation and corresponding Bonferroni-corrected *P* values were reported for direct comparisons (eg NT_50_ for wild-type [WT] spike pseudovirus vs SNAb titer for WT spike; NT_50_ for Alpha spike pseudovirus vs SNAb titer for Alpha spike). Vaccine- and hybrid-immune titer were used to quantify the correlation between the assays. Pre-COVID-19 pandemic serum showed no quantifiable NT_50_ or SNAb titers for any analyte, and was not used for the correlation assay.

##### Predicted neutralization breadth score

The breadth of neutralization capacity against spikes was quantified similarly to Chen et al.^48^ In brief, all of the SNAb titers were summed up as a total titer. An equation is shown as: $\sum_{i=WT\;}^{\mathrm{All}\;\mathrm{Strains}}\;{\mathrm{SNAB}}_{i}$. All strains included WT and all other lineages in this tested array: Alpha, Beta, Delta, BA.1, BA.2, BA.5, BQ.1.1, JN.1, XBB.1.5, and KP.3. The resulting sum for each specimen was scored and plotted.

##### Volcano plot correlations

Systems serology binding features were correlated with WT and composite spike SNAb titers using a Pearson correlation. Resulting *R* values were tabulated, as was the false discovery rate (FDR)–adjusted *P* value for pairwise correlations. The values were plotted using thresholds for significance being |*R*| ≥ 0.75 and FDR *P* value <0.05. Features achieving significance were labeled, while all other features were left gray. Individual dots on the volcano plot were colored based on the antibody feature (total IgG, IgG1, IgM, FcγR2A, etc). All plotting and analysis was done using R Studio.

##### Bland–Altman method agreement analysis

The SNAb assay was assessed for results/performance agreement with pseudovirus NT_50_ values to the spikes using a Bland–Altman method agreement analysis. We used a similar approach to Heeringa et al., who employed the Bland–Altman method agreement analysis to interrogate the performance of microneutralization titer and hemagglutination inhibition assay.^49^ The mean titer between the 2 assays, each in log_10_, was quantified and the difference in paired titers for each specimen (log_10_) was quantified. A difference of 0 would indicate that each assay arrived at exactly the same titer. All specimens were analyzed simultaneously, regardless of the number of antigen exposures and time since antigen encounter. This was also done across immune groups (hybrid or vaccine immune). Pre-COVID serum samples were not used for this analysis as titers were beneath the limit of detection for both NT_50_ and SNAb assays for all shared spike variants.

For all variants assayed, 95% confidence intervals (CIs) were calculated based on the mean differences between the 2 assays for each paired event. Given that the difference was calculated as being the NT_50_ value − SNAb value, a positive mean bias score would indicate that the pseudovirus NT_50_ values were higher, and likewise, a negative bias score indicates that the SNAb values were higher. All calculations and plots were done using R Studio. Further information on Bland–Altman method agreement analyses can be found in previous publications.^49^^,^^70^

## Results

### Development of a multiplex-based predicted neutralization readout for SARS-CoV-2

We initially sought to create a high-throughput, multiplex-based neutralization platform using SARS-CoV-2 variant spikes, from ancestral (or WT) to highly diverged Omicron sublineages. We termed this approach a systems-predicted neutralizing antibody (SNAb) titer assay. We immobilized variant spikes onto magnetic beads and validated their binding to the human SARS-CoV-2 receptor ACE2. Increasing concentrations of ACE2 yielded a canonical binding:substrate profile to the various spikes (Fig. S1A). We deliberately chose ancestral (WT) spike (also known as Wu-1 spike), Delta spike, and KP.3 spike to capture the breadth of spike variants binding to ACE2. The binding of these SARS-CoV-2 spike variants was exclusive to ACE2 as there was no appreciable binding to human CD4, the HIV-1 co-receptor, even at saturating conditions of the receptor. As a separate control, we found that ACE2 binding in this system was specific to the SARS-CoV-2 spikes and was not observed for other virus receptor-binding proteins, such as influenza HA (lineage H1N1 A/Wisconsin/2022), HCMV Gb, or Ebola GP (Fig. S1B). Likewise, none of these receptor binding–negative controls showed any binding to human CD4 (Fig. S1B).

**Figure 1.**
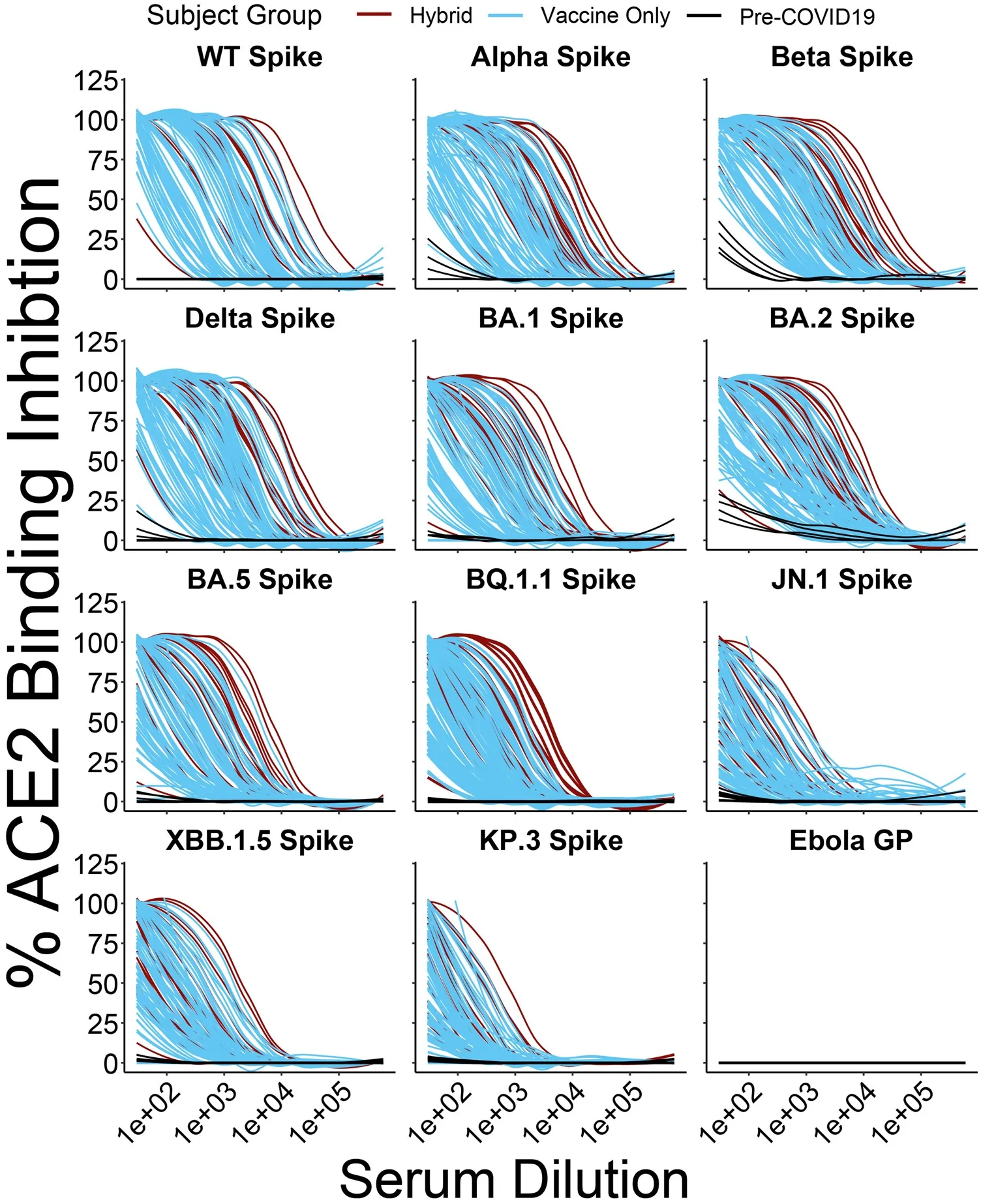
Multiplex binding inhibition of SARS-CoV-2 spike variants and the human receptor ACE2. Binding inhibition curve fits for pre-COVID-19 (dark gray), vaccine-immune (blue), and hybrid-immune (red) serum samples to an array of SARS-CoV-2 spike variant trimers; Ebola GP was used as a negative ACE2 binding control. Each colored line represents the binding inhibition of spike to human ACE2 at the indicated serum dilution (*x* axis). The *y* axis represents the % inhibition. Individual samples were done in technical replicates, and mean values for each dilution were used to generate the inhibition curve. The color legend is shown at the top. All analytes were measured simultaneously.

We then assessed the inhibition of spike variants binding to ACE2 using this system. We profiled this binding inhibition using serum samples from a cohort of mRNA-vaccinated and hybrid-immune participants. As negative controls, we also used specimens from naïve participants taken before the COVID-19 pandemic began (pre-COVID-19 samples) (Table 2). Binding of the spike variants to ACE2 in the presence of serially diluted serum samples was assessed (Fig. S2). From these binding assays, we plotted fits (see Materials and Methods) of the percent inhibition of ACE2 binding to the spike variants, with 100% being complete antibody-mediated inhibition and 0% being the complete absence of any inhibition. As a control, we also quantified the inhibition of Ebola GP binding to ACE2. Pre-COVID-19 serum samples (dark gray lines) did not show appreciable inhibition to any of the spike variants tested, even at high serum concentrations. In contrast, hybrid-immune (red) and vaccine-only (blue) participants showed varying degrees of inhibition to the spikes (Fig. 1).

**Table 2.** Cohort overview of time since most recent antigen encounter.

	Timepoint 1	Timepoint 2	Timepoint 3	Timepoint 4	Timepoint 5
**Total**	15	34	33	3	21
**Vaccine only, 1 dose**	Day 22 (15)	…	…	…	…
**Vaccine only, 2 doses**	Day 28 (10)	Day 78 (7)	Day 105 (10)	Day 223 (6)	…
**Vaccine only, 3 doses**	Day 17 (6)	Day 43 (5)	Day 82 (10)	Day 114 (8)	Day 148 (4)
**Hybrid, 1 dose**	Day 22 (3)	…	…	…	…
**Hybrid, 2 doses**	Day 28 (5)	Day 78 (4)	Day 105 (5)	Day 223 (7)	…

Parentheses number next to the days indicate the number of samples at that given time point.

**Figure 2.**
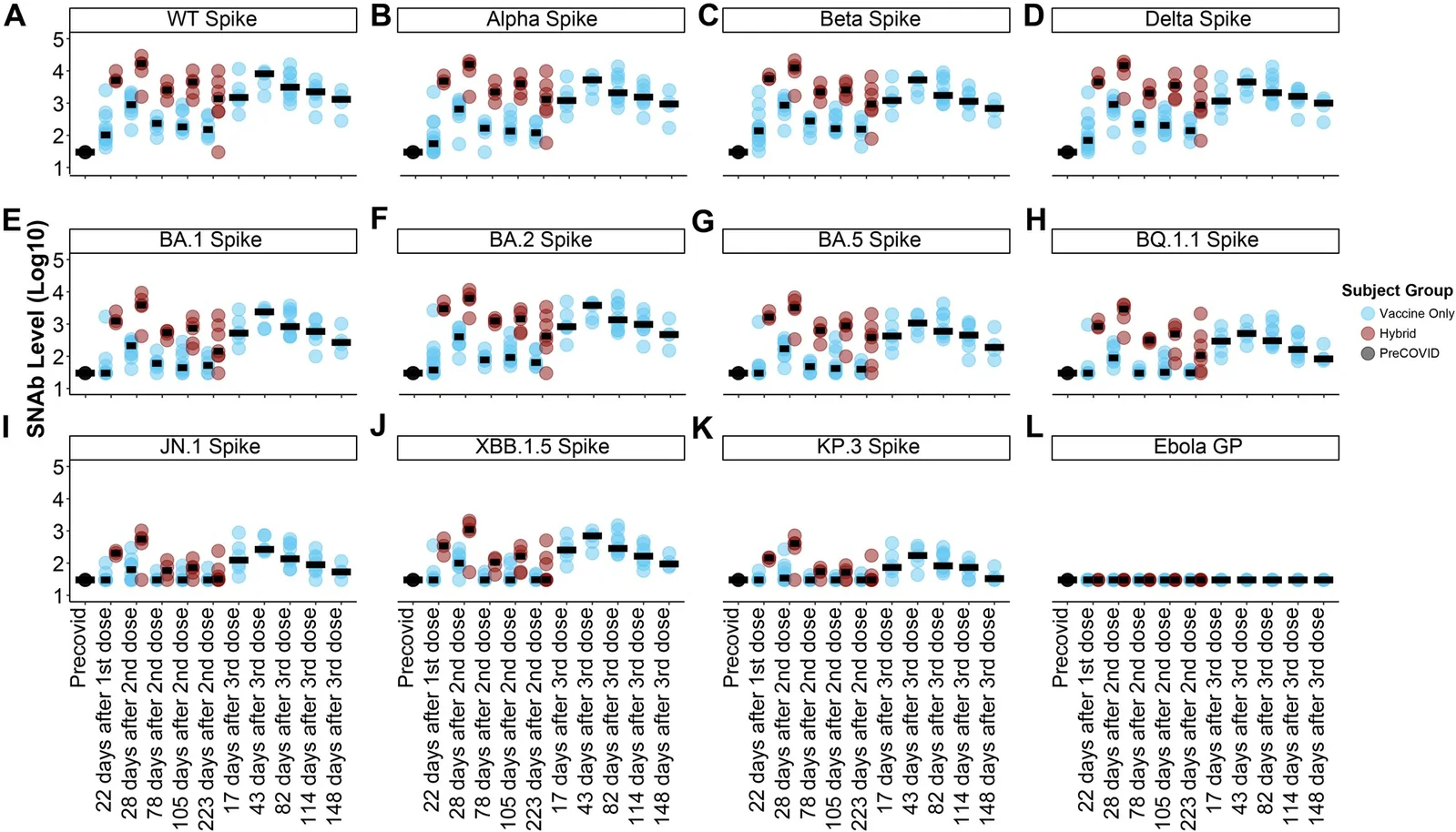
Quantified systems-predicted neutralizing antibody (SNAb) titers to SARS-CoV-2 spikes show exposure and waning sensitive dynamics. (A) Reciprocal serum dilutions for 50% ACE2 binding inhibition (SNAb titer) were quantified for the SARS-CoV-2 WT spike trimer. Shown on the *x* axis are the vaccine doses received and the timeframe after the most recent vaccination. Shown on the *y* axis is the reciprocal SNAb titer in log_10_. Each circle represents the SNAb titer of an individual participant (mean of technical replicates); in blue are vaccine-only participants, in red are hybrid participants, and in dark gray is pre-COVID-19 serum. The median of each group at each time point is shown in a horizontal black bar. (B–K) Same as (A), but for the indicated SARS-CoV-2 variants (all trimers). (L) Same as (A), but for the negative control Ebola GP. Shown on the right is the color legend.

From the binding inhibition curve fits generated in Fig. S2 and Fig. 1, we quantified the 50% inhibition levels via reciprocal serum dilution titer. This value represents the SNAb titer. Samples were colored by the type of immunity assayed (blue circles, vaccine; red circles, hybrid; dark gray circles, pre-COVID-19 serum), number of antigen encounters, and days after antigen encounter were shown. This allows for longitudinal profiling of how SNAb titers moved, and if this movement was dependent on the variant. SNAb titers were highest for WT spike showed a typical priming, boosting, and waning dynamic (Fig. 2A). All hybrid-immune participants were infected prior to vaccination, and therefore 22 days after the first dose represents a total of 2 antigen encounters (infection, then vaccination). It was expected that SNAb titers would be higher than the vaccine-only group. Hybrid-immune participants in this cohort did not receive a third dose, so the right-hand side of each panel exclusively quantifies SNAb titers to WT spike in vaccine-only participants (blue circles). No WT spike SNAb titers from pre-COVID-19 sera were quantifiable.

The same approach was then used to quantify a SNAb titer for the remaining spike variants. As expected, more diverged spike variants showed considerably less predicted neutralizing antibody levels compared to WT spike. SNAb titers for vaccine-only participants showed low levels for Omicron-lineage spikes that quickly waned to near background levels after 2 doses. It was only after 3 doses of vaccine that SNAb levels persisted for most of these Omicron lineage spikes. For the hybrid-immune group, a second dose of the vaccine (3 cumulative antigen encounters) showed SNAb levels to all variants tested, but those too were subject to a rapid decline for highly diverged Omicron lineages such as JN.1 and KP.3 (Fig. 2B–K). SNAb levels to Ebola GP are shown as a reference (Fig. 2L).

These results demonstrate that SNAb can be performed to an array of spike variants simultaneously, and that variants with predicted neutralization escape showed a considerable reduction in predicted titers through this assay.

### SARS-CoV-2 SNAb correlates with existing neutralization outputs

**Figure 3.**
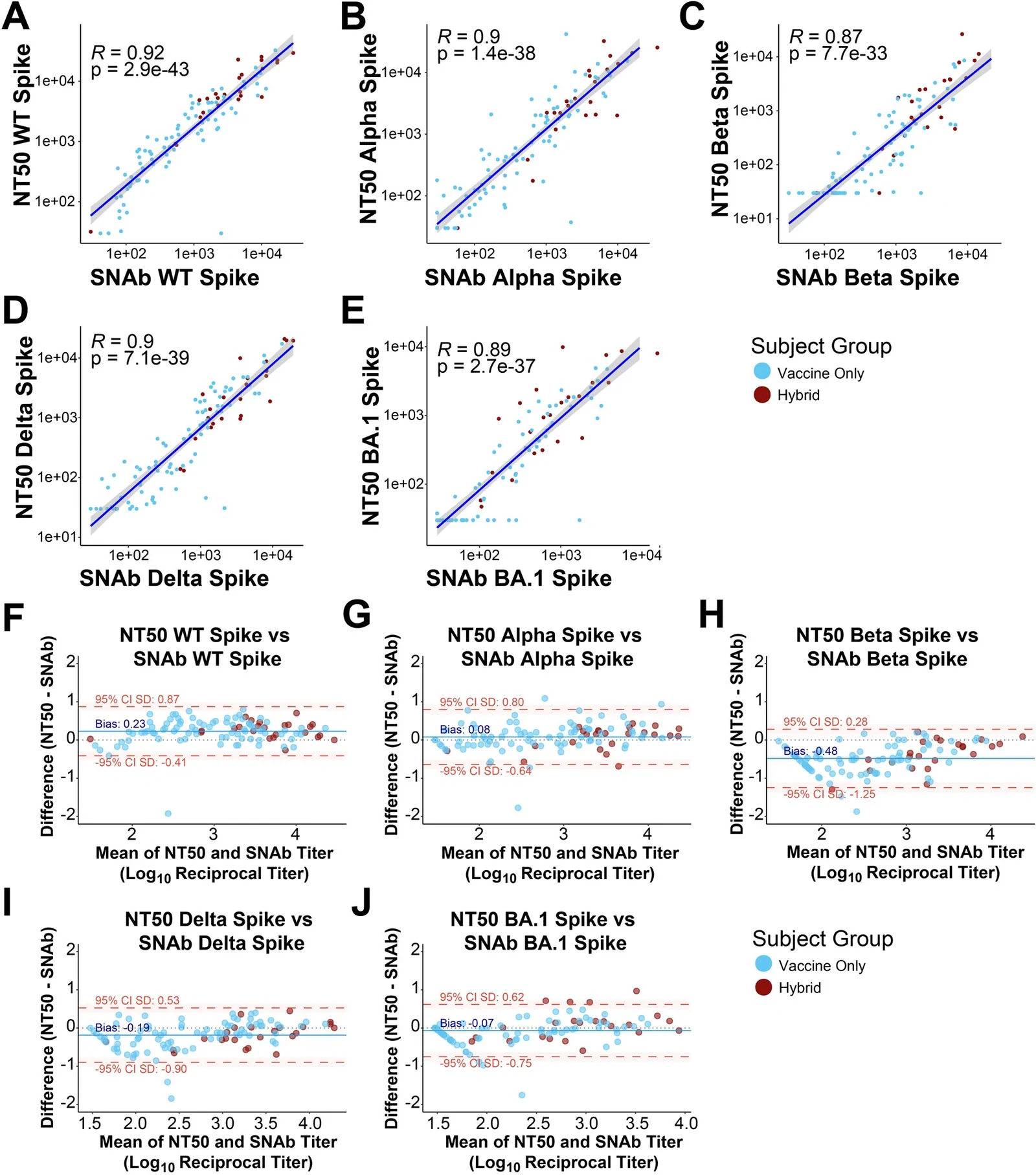
Comparison of SNAb and pseudovirus NT_50_ assays. (A) Correlation between SNAb titers and reported NT_50_ titers for SARS-CoV-2 WT spike. Pseudovirus NT_50_ values were correlated with the calculated SNAb titers for vaccine- and hybrid-immune specimens for WT spike; pre-COVID-19 immune serum was not included in this correlation as neither assay had any detectable titers to WT spike. The Pearson correlation coefficient (*R*) and corresponding Bonferroni-corrected *P* value for the 2 neutralization outputs are shown in the upper left. On the *x* axis is the SNAb WT spike titer, and on the *y* axis is the NT_50_ WT spike titer; all datapoints for each immune group are shown independent of the time since immunization. Shown is the best-fit correlation line (solid blue line) along with the 95% CIs (shaded gray region). (B–E) Same as (A), but correlating SNAb and NT_50_ values for SARS-CoV-2 Alpha spike variant (B), Beta spike variant (C), Delta spike variant (D), and Omicron BA.1 spike variant (E). (F) Bland–Altman method agreement analysis for NT_50_ and SNAb titers to WT spike. Shown on the *x* axis is the mean between NT_50_ and SNAb titers for individual participants, colored by immune type. Shown on the *y* axis is the difference in reported titer between the 2 assays (NT_50_ − SNAb titer in log_10_). The solid horizontal blue line is the mean bias across all samples; the dashed orange lines represent the 95% CI for all samples for WT spike titers. A bias score of 0 would indicate perfect agreement between the 2 assays. A positive bias score indicates a preference for NT_50_, and a negative bias score indicates a preference for SNAb. (G–J) Same as (F), but a Bland–Altman method agreement analysis for NT_50_ and SNAb titers to Alpha spike variant (G), Beta spike variant (H), Delta spike variant (I), and Omicron BA.1 spike variant (J).

To ask how tightly SNAb titers correlated with reported NT_50_ values of the serum samples, we performed a variant-specific direct correlation assay. The reported NT_50_ values for WT, Alpha, Beta, Delta, and Omicron BA.1 spike from Chen et al.^48^ were correlated with the SNAb titer for both vaccine- and hybrid-immune groups. Previously reported neutralizing WT spike antibody titers from this cohort obtained through pseudovirus NT_50_ were correlated to SNAb titers for WT spike. By unbiasedly analyzing all values, regardless of number of exposures and route of immunity, we found that the SNAb titers correlated exceptionally well with reported NT_50_ values (*R* = 0.92, *P* < 2.9E-43) (Fig. 3A).

We further correlated our SNAb titers with existing NT_50_ values reported in Chen et al. for Alpha, Beta, Delta, and Omicron BA.1 spike.^48^ Like the correlation for WT, the SNAb titers tightly correlated with the NT_50_ titers for each of these variants (Fig. 3B–E). Importantly, no significant drop-off in correlation was noted for any of the variants assayed. Collectively, these results show that SNAb results are tightly correlated with conventional neutralization assays such as pseudovirus NT_50_.

To assess if the SNAb and pseudovirus NT_50_ assays yielded comparable results, and not just correlated values, we performed a Bland–Altman method agreement analysis. Here, reciprocal titers from NT_50_ and SNAb assays were averaged (*x* axis), and the difference between the assays (in log_10_) was quantified (*y* axis) to identify if one approach consistently over- or underestimated the titer. A value of 0 would indicate complete agreement. For WT spike, all titers were plotted ambivalent to time since antigen encounter (*N* = 105 total samples). Very tight agreement between NT_50_ and SNAb titers was observed, with the 95% CI of all observations being within 1 order of magnitude. A bias score of 0.23 for WT spike was calculated, indicating that NT_50_ values were slightly higher, on average, than SNAb titers (95% CI, −0.41 to 0.87) (Fig. 3F). The same process was repeated for Alpha spike, Beta spike, Delta spike, and Omicron spike. These were the only spike variants that had NT_50_ values to reference SNAb titers against. Alpha spike had an NT_50_ bias of 0.08 (95% CI, −0.64 to 0.80) (Fig. 3G), Beta spike had a SNAb bias of 0.48 (95% CI, −1.25 to 0.28) (Fig. 3H), Delta spike had a SNAb bias of 0.19 (95% CI, −0.90 to 0.53) (Fig. 3I), and Omicron spike had a SNAb bias of 0.07 (95% CI, −0.75 to 0.62) (Fig. 3J). These values fall in line with high degrees of assay agreement seen in other neutralizing antibody titer assays.^49^

These results showed that neutralizing antibody titers quantified by SNAb are in tight agreement with pseudovirus-based neutralization assays. Additionally, multiplexing the neutralization readout with SNAb titer did not bias the results, nor was there a detectable trend in assay performance based on antigenic drift.

### Incorporation of SARS-CoV-2 SNAb into a systems serology workflow

To assess what antibody features were contributing to neutralizing responses, we performed systems serology binding analyses on the specimens from vaccine-only and hybrid-immune participants. We referenced these binding levels to pre-COVID-19 serum samples, similar to our neutralization output. We arranged all specimens in order of increasing WT NT_50_ values for each group.

**Figure 4.**
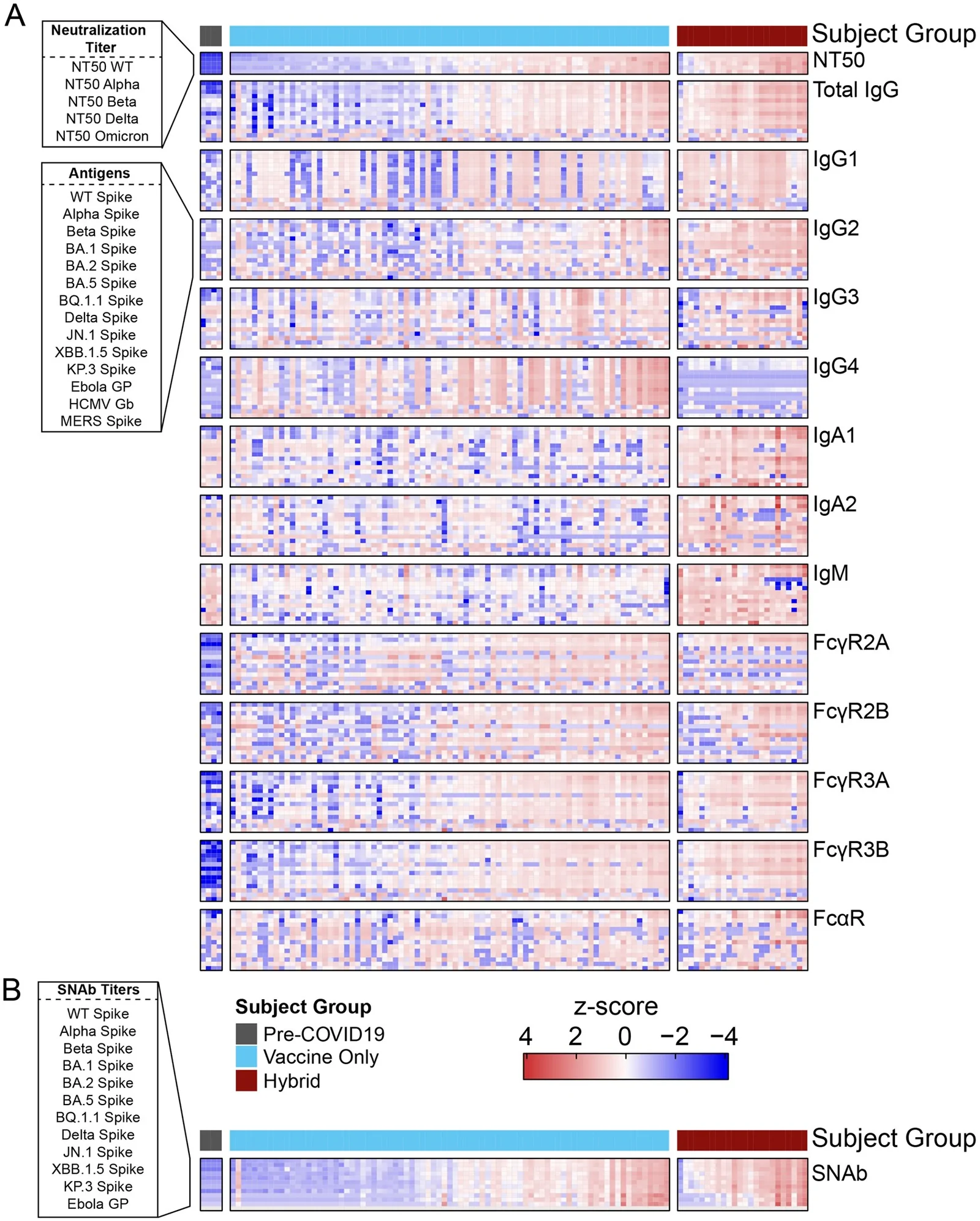
Systems serology binding assays to spike variants and controls. (A) Antibody binding levels to the indicated antigens from human serum samples of pre-COVID-19 pandemic (dark gray), vaccine-immune (blue column), and hybrid-immune (red). Each group was arranged in increasing pseudovirus NT_50_ values to WT spike (top row). All binding levels were log_10_ transformed and each combination of isotype and antigen were independently z-scored to standardize across analytes. The order of targets is shown on the left callout boxes; the antibody feature is shown on the right. Each column represents one participant. (B) SNAb levels for the groups were similarly shown for each immune group. The order of SNAb targets is shown on the left callout box. All SNAb levels were standardized through z-scoring. Color legend is shown on the top, and is the same as (A). All specimens were arranged in the same order as (A), and each column represents one participant.

It was immediately observable that antibody isotypes and subclasses were distinct between the 2 immunity arms. Timepoints and number of antigen exposures were not included on this heatmap as we were interested in how antibody levels trended with NT_50_ values. For vaccine-immune serum samples, IgG4 and FcγR2A levels increased with increasing NT_50_ values. In contrast, hybrid-immune serum samples showed an enrichment for IgA and IgG2 with increasing NT_50_ values (Fig. 4A). These results were scaled. We corroborated these results by plotting them in raw log_10_ values (Fig. S3A).

We further asked if SNAb titers to the array of spike variant trimers also trended with NT_50_ values to WT spike. As expected, SNAb titers to the array of spikes followed the same trend for both vaccine- and hybrid-immune participants. No quantifiable SNAb titers to Ebola GP to ACE2 were present for any participant for any group. Additionally, pre-COVID-19 specimens showed no SNAb titers for all spike variants tested (Fig. 4B). These scaled results were again confirmed through plotting of raw log_10_ values (Fig. S3B).

**Figure 5.**
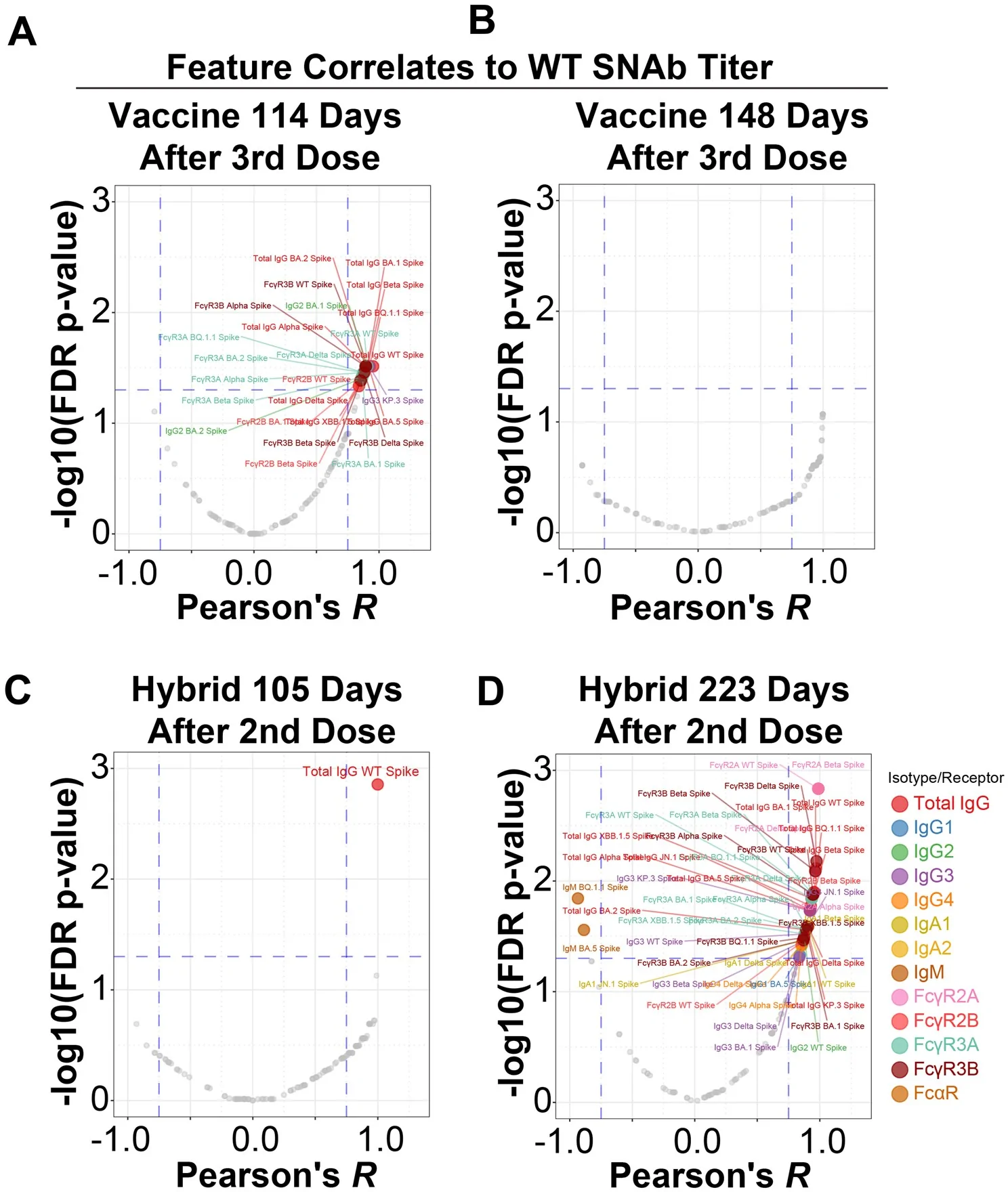
Identification of antibody features correlating with WT spike SNAb titers through systems serology. (A) Pearson correlation coefficients (*R*) for all antibody features against WT spike SNAb titers were plotted using a volcano plot for 3-dose vaccine recipients 114 days after the third dose. Shown on the *x* axis is the *R* value to WT spike SNAb titers, ranging from −1 to 1. Shown on the y-axis is the FDR-adjusted *P* value in -log_10_. Thresholds for significance were an |*R*| ≥ 0.75 and an FDR *P* < 0.05 (dashed blue lines). All features passing these thresholds were highlighted and labeled; all other features were left gray and not labeled. Three antigen encounters, either all by vaccine or hybrid immunity, followed by a 2-dose mRNA vaccine series, were used to build the correlation. (B) Same as (A), but for 3-dose vaccine recipients 148 days after the third dose. Other time points could not be assessed due to low *n* or imbalance in antigen encounters. (C) Same as (A), but for hybrid-immune participants 105 days after receiving their second mRNA vaccine dose (3 total antigen encounters). (D) Same as (C), but for hybrid-immune participants 223 days after receiving their second mRNA vaccine dose (3 total antigen encounters). Other time points could not be assessed due to low *n* or imbalance in antigen encounters. The color legend for the antibody features for all volcano plots is shown on the right.

We used these results to find which antibody features were correlated with SNAb titers. We created volcano plots to identify antibody features with a strong Pearson correlation coefficient (*R *≥ 0.75) and an FDR-adjusted *P* < 0.05. The FDR adjustment was made to ensure that significant features were identified while adjusting for the large number of comparisons being made through our approach. Participants who received 3 doses of a vaccine or hybrid-immune participants who received 2 vaccines were used for this comparison. We selected time frames >80 days after the latest antigen encounter to identify features correlated with WT spike SNAb titers. This was done as several earlier timepoints did not have as many collections, and also to understand how the antibody repertoire settles after peak titers. For SNAb to WT spike for vaccine-immune participants (3 doses) 114 days after the final dose, a number of features were significantly correlated with WT SNAb titers (|*R*| ≥0.75, FDR-adjusted *P* < 0.05). These features included total IgG, IgG2, IgG3, FcγR2B, FcγR3A, and FcγR3B binding antibodies to a diverse array of spike variants, including WT, Alpha, Beta, Delta, BA.1, BA.2, BQ.1.1, XBB.1.5, and KP.3 spike (Fig. 5A). However, by day 148, no correlates of SNAb titers to WT spike meeting the thresholds were present (Fig. 5B). This may have been due to the limited number of participants’ serum samples we had for this timepoint. In support of this, numerous correlates to WT spike SNAb titers were identified at day 82 post–third dose (Fig. S4A). This timepoint had a higher *n* for this immune group. All features correlated with WT spike SNAb titers for vaccine-immune participants at these timepoints are shown in Table S2.

For hybrid-immune participants who had received their completed vaccine series (2 doses after infection and recovery), only total IgG to WT spike was a significant correlate to WT spike SNAb titers 105 days after the second vaccination (Fig. 5C). Interestingly, though, at 223 days after the second dose of the mRNA vaccine series, a large number of features achieved statistical significance both positively and negatively to WT spike SNAb titers (Fig. 5D). Negative correlates of WT spike SNAb titers were exclusively IgM features (BA.5 spike and BQ.1.1 spike). This is not entirely unexpected as IgM is predominantly non-affinity matured and wanes with time since initial antigen encounter,^50^^,^^51^ and is usually not recalled like affinity-matured isotypes like IgA and IgG.^47^^,^^48^ The features positively correlating with WT spike SNAb titers were total IgG, IgG2, IgG3, IgG4, IgA1, FcγR2A, FcγR2B, FcγR3A, and FcγR3B to an array of spike trimers. All features correlated with WT spike SNAb titers for the hybrid-immune participants at these timepoints are shown in Table S3.

**Figure 6.**
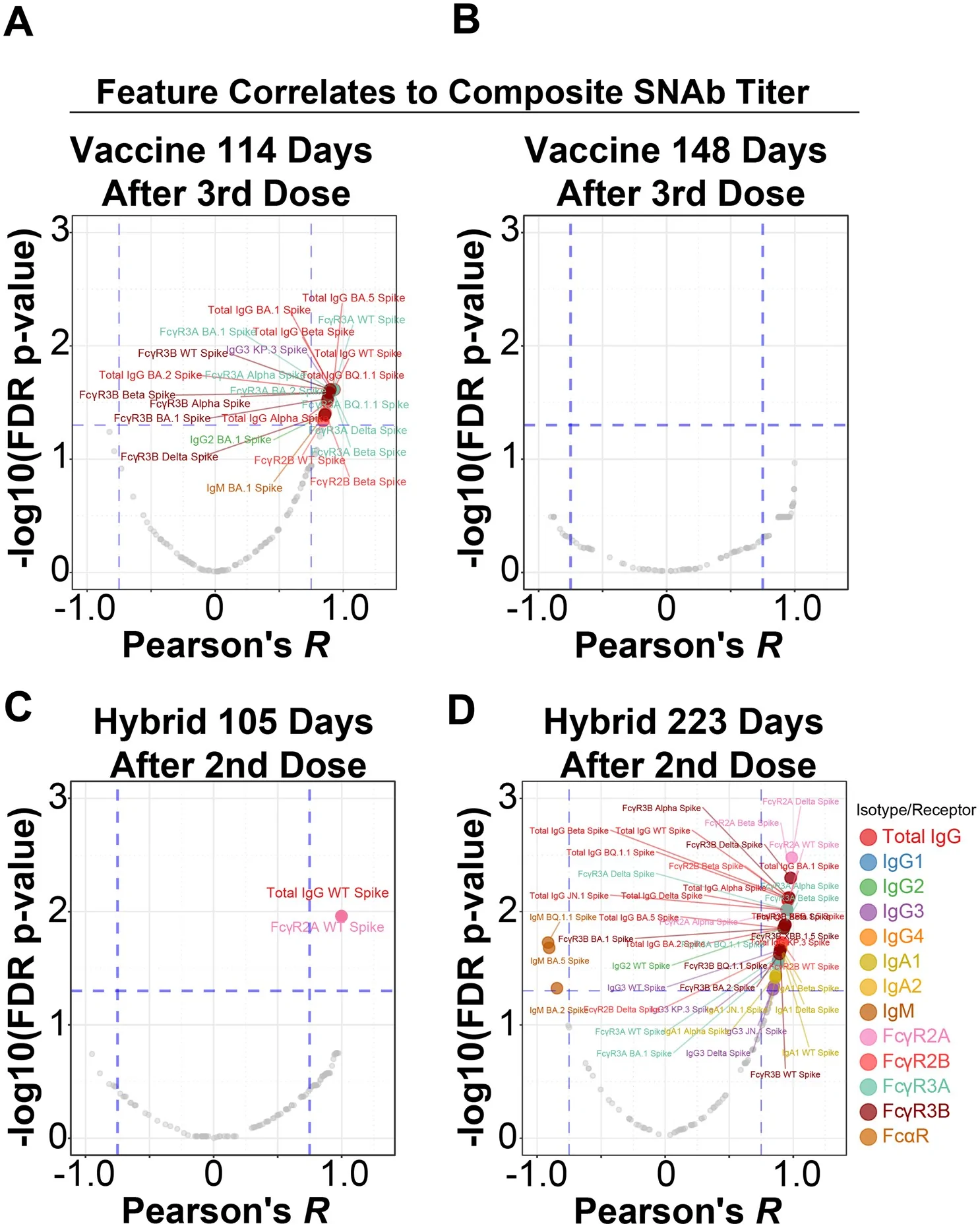
Identification of antibody features correlating with composite SNAb titers through systems serology. (A) Pearson correlation coefficients (*R*) for all antibody features against composite spike SNAb titers were plotted using a volcano plot for 3-dose vaccine recipients 114 days after the third dose. Shown on the *x* axis is the *R* value to composite spike SNAb titers (sum of all spike SNAb titers), ranging from −1 to 1. Shown on the *y* axis is the FDR-adjusted *P* value in -log_10_. Thresholds for significance were an |*R*| ≥ 0.75 and an FDR *P* value <0.05 (dashed blue lines). All features passing these thresholds were highlighted and labeled; all other features were left gray and not labeled. Three antigen encounters, either all by vaccine or hybrid immunity followed by a 2-dose mRNA vaccine series, were used to build the correlation. (B) Same as (A), but for 3-dose vaccine recipients 148 days after the third dose to composite SNAb titers. Other time points could not be assessed due to low *n* or imbalance in antigen encounters. (C) Same as (A), but for hybrid-immune participants 105 days after receiving their second mRNA vaccine dose to composite SNAb titers (3 total antigen encounters). (D) Same as (C), but for hybrid-immune participants 223 days after receiving their second mRNA vaccine dose (3 total antigen encounters). Other time points could not be assessed due to low *n* or imbalance in antigen encounters. The color legend for the antibody features for all volcano plots is shown on the right.

The same process was applied to antibody features correlating with composite SNAb titers for vaccine- and hybrid-immune participants. Log-transformed SNAb titers for the vaccine immune group at days 114 and 148 post–third dose were summed for all spike trimers, and features significantly correlating with the sum were identified through a volcano plot. At day 114 post–third dose, several features showed significant correlation with composite SNAb titers, including total IgG, IgG3, IgM, FcγR2B, FcγR3A, and FcγR3B binding antibodies to various spikes (Fig. 6A). No features significantly correlating with composite SNAb titers at day 148 post–third dose were identified (Fig. 6B). Again, this may have been due to the limited number of participants’ serum samples available to us for this timepoint as numerous correlates for composite SNAb titers were observed at day 82 (Fig. S4B). For the hybrid-immune group, 105 days after the second dose, only total IgG and FcγR2A to WT spike were identified as being statistically significantly correlated with composite SNAb titers (Fig. 6C). In contrast, numerous features were identified as being significantly correlated with composite SNAb titers at day 223 post–second dose in the hybrid-immune group. This included some features that were inversely correlated with composite SNAb titers, such as IgM to BA.2, BA.5, and BQ.1.1 spike. Positively correlating features included total IgG, IgG2, IgG3, IgA1, FcγR2A, FcγR2B, FcγR3A, and FcγR3B binding antibodies to a diverse array of spike variants (Fig. 6D). Features correlated with composite spike SNAb titers for the immune groups are shown in Tables S2 and S3.

## Discussion

Neutralizing antibodies have been shown to play a foundational role in limiting disease states caused by respiratory viruses. Neutralizing antibody titer is a validated correlate of protection against several viral pathogens, including the recently emerged SARS-CoV-2.^21–25^ However, as viruses spread throughout the population, resistance to neutralization can occur, which prompts deployment of vaccine boosters meant to provide enhanced neutralization breadth.^26–28^^,^^50^^,^^52–54^ To that end, rapidly quantifying neutralization breadth and identifying factors contributing to the underlying neutralization phenotype are important not just for SARS-CoV-2, but other vaccine-targeted diseases.

Here, we developed an assay to quantify and characterize neutralization breadth at the systems level. This assay, which we term systems-predicted neutralizing antibody (SNAb) titer assay, can predict the neutralizing antibody potential to dozens of analytes simultaneously. While it is not itself a cell-based neutralization assay, it models the antibody-based inhibition of a receptor-binding protein. The resulting titers aligned closely with pseudovirus-based neutralization assays (Bonferroni-adjusted *P* value <1E-32) for SARS-CoV-2 spike, and the assay itself does not appear to bias the predicted titer compared to pseudovirus NT_50_. The SNAb assay can be further incorporated into a systems serology pipeline to identify features correlating with the neutralization phenotype. We established our approach using both vaccine-only and hybrid-immune specimens taken from human donors. Our results are in agreement with previous reports that hybrid-immune profiles are unique compared to vaccine-only and infection-only phenotypes, and can be linked with enhanced protection.^16–20^^,^^48^^,^^55^^,^^56^ Of course, this is dependent on a positive outcome to the infection and recovery.

This report adds to the growing body of literature that Fc domains of antibodies may play a previously unheralded role in potentiating and broadening neutralization phenotypes.^1^^,^^57–62^ Both vaccine- and hybrid-immune responses had neutralization correlates, both to the WT spike and to broadly neutralizing phenotypes, leveraged by FcγR-binding antibodies. Interestingly, IgG2, IgG3, and IgG4, along with IgA1, were also part of these neutralization correlates to SARS-CoV-2 spike. This supports a model whereby neutralizing antibodies exist in polyclonal swarms and may be further influenced by the compartment of antigen encounter.^63–65^ Additionally, the strong influence of FcγR binding antibodies supports the model that the Fc domain of antibodies plays an important role in coordinating neutralizing antibody responses.

In summary, we have developed a pipeline to quantify predicted neutralization capacity and breadth at the systems scale. Our results are in tight correlation with existing neutralizing antibody quantification assays used for regulatory approvals, but do not require higher biosafety level containment and can be completed within 1 day, compared to typical neutralization assays that can take multiple days to a week. This approach estimated neutralization titer values without over- or underestimating the titer compared with NT_50_. This multiplexed-based neutralization assay can further be incorporated into systems serology workflows and can identify signatures of neutralization at the isotype and subclass level.

### Limitations of this study

There are several limitations to this study that must be acknowledged. The cohort analyzed was not powered to account for confounding variables such as sex, age, body mass index, and others. Cohorts with a higher number of tracked individuals over time, and balanced for a number of demographic features, are needed for future studies to characterize how they exert influence on direct-target and target-related neutralization phenotypes. We also acknowledge that we did not have an infection-only group to perform our assays on. We have previously shown that vaccination after infection and recovery enhances immune responses that cannot be explained by additive effects (ie synergistic).^16^ Future studies analyzing how confounders such as age, sex, and infection severity influence neutralization breadth, and how this breadth is expanded by vaccination, would help clarify the stark differences observed between vaccine- and hybrid-immune neutralization and binding profiles. Last, whether protein-based receptors like ACE2 and CD4 behave like sialic acids through this approach is not understood.

## Supplementary Material

vkag106_Supplementary_Data

## Data Availability

Raw data used for this paper have been deposited on the Harvard School of Public Health Systems Serology GitHub page under the accession number QW20250901. All data associated with this study are included in the main manuscript file or the supplementary material. The data, code, and materials used in this study will be made available upon request and execution of a materials transfer agreement, data use agreement, and/or institutional licensing agreement. Correspondence for these should be sent to Ryan P. McNamara (rmcnamara@hsph.harvard.edu).
