# Supplementary material for: A multiplexed, systems-based approach for prediction of antibody neutralization breadth for soluble human receptors
Source: J Immunol. 2026 May 25;215(5):vkag106. doi: 10.1093/jimmun/vkag106 (PMC13200002; doi:10.1093/jimmun/vkag106)
Supplement: vkag106_Supplementary_Data [file vkag106_supplementary_data.pdf]

Supplementary Information

Supplementary Information contains Supplementary Tables 1-3, Supplementary Figures 1-4, and their corresponding Figure Legends

Supplementary Table 1

Reagent	Manufacturer	Identifier
Antibodies		
Anti-human Total IgG-PE	Southern Biotech	9040-09
Anti-human IgG1-PE	Southern Biotech	9052-09
Anti-human IgG2-PE	Southern Biotech	9060-09
Anti-human IgG3-PE	Southern Biotech	9210-09
Anti-human IgG4-PE	Southern Biotech	9200-05
Anti-human IgA1-PE	Southern Biotech	9130-09
Anti-human IgA2-PE	Southern Biotech	9140-09
Anti-human IgM-PE	Southern Biotech	9020-09
Chemicals, peptides, and recombinant proteins		
Human FcγRIIA	Duke University	Custom Order
Human FcγRIIB	Duke University	Custom Order
Human FcγRIIIA	Duke University	Custom Order
Human FcγRIIIB	Duke University	Custom Order
Streptavidin-R-Phycoerythrin, 5 mg	Agilent	PJ31S-5
Sulfo-NHS	Thermo Fisher	106627-54-7
Sulfo-NHS-LC-LC-Biotin	Thermo Fisher	A35358
EDC, no weigh	Pierce	25952-53-8
Tween-20	Fisher Scientific	BP337-100
Bovine Serum Albumin	Millipore Sigma	A4737
WT Spike	Sino Biological	40589-V08H4
Alpha Spike	Sino Biological	40589-V08H12
Beta Spike	Sino Biological	40589-V08H13
BA.1 Spike	Sino Biological	40589-V08H26
BA.2 Spike	Sino Biological	40589-V08H28
BA.5 Spike	Sino Biological	40589-V08H32
BQ.1.1 Spike	Sino Biological	40589-V08H41
Delta Spike	Sino Biological	40589-V08H10

JN.1 Spike	Sino Biological	40589-V08H59
XBB.1.5 Spike	Sino Biological	40589-V08H45
KP.3 Spike	Sino Biological	40589-V08H64
EBOV GP (subtype Zaire)	Sino Biological	40459-V08H
HCMV Glycoprotein B	Sino Biological	10202-V08H1
MERS-Coronavirus Spike	Sino Biological	40069-V08B
Human ACE2	Sino Biological	10108-H08B
Commercial Assays and Kits		
BirA500: BirA biotin-protein ligase standard reaction kit	Avidity	EC 6.3.4.15
xMAP 10X Sheath Concentrate	Thermo Fisher	4050023
Software and Algorithms		
R Studio V 6.0	R Project for Statistical Computing	RRID:SCR_000432
Other		
384-well HydroSpeed Plate Washer	Tecan	30190112
iQue Screener Plus	Intellicyt/Sartorius	11811
Luminex MagPlex-C Microspheres	Fisher Scientific	NC2186992
Luminex xMAP INTELLFLEX	Luminex/Diasorin	APX2020
384-well Plates (non-binding)	Greiner Bio-One	781900

Supplementary Table 2

Target	Correlate Feature	R value	FDR p-value	Days Post 3rd Dose
WT Spike SNAb	Total IgG WT Spike	0.952724	0.030614022	114
WT Spike SNAb	Total IgG Alpha Spike	0.894159	0.030614022	114
WT Spike SNAb	Total IgG Beta Spike	0.908339	0.030614022	114
WT Spike SNAb	Total IgG BA.1 Spike	0.907182	0.030614022	114
WT Spike SNAb	Total IgG BA.2 Spike	0.906671	0.030614022	114
WT Spike SNAb	Total IgG BA.5 Spike	0.91671	0.030614022	114
WT Spike SNAb	Total IgG BQ.1.1 Spike	0.924371	0.030614022	114
WT Spike SNAb	Total IgG Delta Spike	0.849822	0.041483895	114
WT Spike SNAb	Total IgG XBB.1.5 Spike	0.839638	0.046247962	114
WT Spike SNAb	IgG2 BA.1 Spike	0.89879	0.030614022	114
WT Spike SNAb	IgG2 BA.2 Spike	0.858968	0.039549397	114
WT Spike SNAb	IgG3 KP.3 Spike	0.921543	0.030614022	114
WT Spike SNAb	FcγR2B WT Spike	0.862615	0.03849826	114
WT Spike SNAb	FcγR2B Beta Spike	0.845327	0.043353425	114
WT Spike SNAb	FcγR2B BA.1 Spike	0.850507	0.041483895	114
WT Spike SNAb	FcγR3A WT Spike	0.90695	0.030614022	114
WT Spike SNAb	FcγR3A Alpha Spike	0.877275	0.034896578	114
WT Spike SNAb	FcγR3A Beta Spike	0.863402	0.03849826	114
WT Spike SNAb	FcγR3A BA.1 Spike	0.868016	0.038088409	114
WT Spike SNAb	FcγR3A BA.2 Spike	0.874432	0.034903886	114
WT Spike SNAb	FcγR3A BQ.1.1 Spike	0.877037	0.034896578	114
WT Spike SNAb	FcγR3A Delta Spike	0.890523	0.030632303	114
WT Spike SNAb	FcγR3B WT Spike	0.896557	0.030614022	114
WT Spike SNAb	FcγR3B Alpha Spike	0.893509	0.030614022	114
WT Spike SNAb	FcγR3B Beta Spike	0.854655	0.041181938	114
WT Spike SNAb	FcγR3B Delta Spike	0.882424	0.034896578	114
Composite Spike SNAb	Total IgG WT Spike	0.927778	0.024300138	114
Composite Spike SNAb	Total IgG Alpha Spike	0.874525	0.031162781	114
Composite Spike SNAb	Total IgG Beta Spike	0.918755	0.024300138	114
Composite Spike SNAb	Total IgG BA.1 Spike	0.911319	0.024300138	114
Composite Spike SNAb	Total IgG BA.2 Spike	0.896276	0.024300138	114
Composite Spike SNAb	Total IgG BA.5 Spike	0.920465	0.024300138	114
Composite Spike SNAb	Total IgG BQ.1.1 Spike	0.931484	0.024300138	114
Composite Spike SNAb	IgG2 BA.1 Spike	0.853016	0.04068664	114
Composite Spike SNAb	IgG3 KP.3 Spike	0.901287	0.024300138	114
Composite Spike SNAb	IgM BA.1 Spike	0.854473	0.04068664	114
Composite Spike SNAb	FcγR2B WT Spike	0.844357	0.045979761	114
Composite Spike SNAb	FcγR2B Beta Spike	0.861907	0.039074949	114
Composite Spike SNAb	FcγR3A WT Spike	0.923838	0.024300138	114
Composite Spike SNAb	FcγR3A Alpha Spike	0.897277	0.024300138	114
Composite Spike SNAb	FcγR3A Beta Spike	0.906181	0.024300138	114
Composite Spike SNAb	FcγR3A BA.1 Spike	0.899804	0.024300138	114
Composite Spike SNAb	FcγR3A BA.2 Spike	0.885083	0.02697864	114
Composite Spike SNAb	FcγR3A BQ.1.1 Spike	0.924654	0.024300138	114
Composite Spike SNAb	FcγR3A Delta Spike	0.91347	0.024300138	114
Composite Spike SNAb	FcγR3B WT Spike	0.899616	0.024300138	114
Composite Spike SNAb	FcγR3B Alpha Spike	0.890839	0.025888556	114
Composite Spike SNAb	FcγR3B Beta Spike	0.889033	0.025888556	114
Composite Spike SNAb	FcγR3B BA.1 Spike	0.879664	0.029132695	114
Composite Spike SNAb	FcγR3B Delta Spike	0.858019	0.040323039	114

Table S2. Antibody features significantly correlated with SNAb titers from vaccine-only immunity.

Supplementary Table 3

Target	Correlate Feature	R value	FDR p-value	Days Post 2nd Dose
WT Spike SNAb	Total IgG WT Spike	0.999551	0.00139458	105
Composite Spike SNAb	Total IgG WT Spike	0.998076	0.010958199	105
Composite Spike SNAb	FcγR2A WT Spike	0.99718	0.010958199	105
WT Spike SNAb	Total IgG WT Spike	0.988431	0.001467147	223
WT Spike SNAb	Total IgG Alpha Spike	0.922293	0.018448703	223
WT Spike SNAb	Total IgG Beta Spike	0.954979	0.012596677	223
WT Spike SNAb	Total IgG BA.1 Spike	0.969492	0.007678834	223
WT Spike SNAb	Total IgG BA.2 Spike	0.888509	0.027576247	223
WT Spike SNAb	Total IgG BA.5 Spike	0.92241	0.018448703	223
WT Spike SNAb	Total IgG BQ.1.1 Spike	0.952696	0.012655295	223
WT Spike SNAb	Total IgG Delta Spike	0.90706	0.025797863	223
WT Spike SNAb	Total IgG JN.1 Spike	0.928192	0.016792841	223
WT Spike SNAb	Total IgG XBB.1.5 Spike	0.934067	0.014366091	223
WT Spike SNAb	Total IgG KP.3 Spike	0.897548	0.026302773	223
WT Spike SNAb	IgG1 BA.5 Spike	0.840097	0.046828639	223
WT Spike SNAb	IgG2 WT Spike	0.875683	0.032162693	223
WT Spike SNAb	IgG3 WT Spike	0.880368	0.030100016	223
WT Spike SNAb	IgG3 Beta Spike	0.862987	0.035169711	223
WT Spike SNAb	IgG3 BA.1 Spike	0.835681	0.048983173	223
WT Spike SNAb	IgG3 Delta Spike	0.857443	0.037094822	223
WT Spike SNAb	IgG3 JN.1 Spike	0.899807	0.025797863	223
WT Spike SNAb	IgG3 KP.3 Spike	0.920673	0.018526163	223
WT Spike SNAb	IgG4 Alpha Spike	0.854239	0.03824597	223
WT Spike SNAb	IgG4 Delta Spike	0.857343	0.037094822	223
WT Spike SNAb	IgA1 WT Spike	0.905633	0.025797863	223
WT Spike SNAb	IgA1 Beta Spike	0.901804	0.025797863	223
WT Spike SNAb	IgA1 Delta Spike	0.863102	0.035169711	223
WT Spike SNAb	IgA1 JN.1 Spike	0.868597	0.034235657	223
WT Spike SNAb	IgM BA.5 Spike	-0.887	0.027679152	223
WT Spike SNAb	IgM BQ.1.1 Spike	-0.93531	0.014366091	223
WT Spike SNAb	FcγR2A WT Spike	0.987221	0.001467147	223
WT Spike SNAb	FcγR2A Alpha Spike	0.900658	0.025797863	223
WT Spike SNAb	FcγR2A Beta Spike	0.994402	0.000561194	223
WT Spike SNAb	FcγR2A Delta Spike	0.965673	0.008161056	223
WT Spike SNAb	FcγR2B WT Spike	0.864017	0.035169711	223
WT Spike SNAb	FcγR2B Beta Spike	0.94809	0.013262368	223
WT Spike SNAb	FcγR3A WT Spike	0.936462	0.014366091	223
WT Spike SNAb	FcγR3A Alpha Spike	0.895497	0.026687436	223
WT Spike SNAb	FcγR3A Beta Spike	0.944088	0.013262368	223
WT Spike SNAb	FcγR3A BA.1 Spike	0.890259	0.027576247	223
WT Spike SNAb	FcγR3A BA.2 Spike	0.888577	0.027576247	223
WT Spike SNAb	FcγR3A BQ.1.1 Spike	0.942354	0.013262368	223
WT Spike SNAb	FcγR3A Delta Spike	0.936875	0.014366091	223
WT Spike SNAb	FcγR3A XBB.1.5 Spike	0.880345	0.030100016	223
WT Spike SNAb	FcγR3B WT Spike	0.94745	0.013262368	223
WT Spike SNAb	FcγR3B Alpha Spike	0.943894	0.013262368	223
WT Spike SNAb	FcγR3B Beta Spike	0.964195	0.008161056	223
WT Spike SNAb	FcγR3B BA.1 Spike	0.891873	0.027576247	223
WT Spike SNAb	FcγR3B BA.2 Spike	0.868389	0.034235657	223
WT Spike SNAb	FcγR3B BQ.1.1 Spike	0.873797	0.032506108	223
WT Spike SNAb	FcγR3B Delta Spike	0.973728	0.006620462	223

WT Spike SNAb	FcγR3B XBB.1.5 Spike	0.902698	0.025797863	223
Composite Spike SNAb	Total IgG WT Spike	0.964614	0.007528919	223
Composite Spike SNAb	Total IgG Alpha Spike	0.957431	0.007974753	223
Composite Spike SNAb	Total IgG Beta Spike	0.961644	0.007528919	223
Composite Spike SNAb	Total IgG BA.1 Spike	0.969618	0.007528919	223
Composite Spike SNAb	Total IgG BA.2 Spike	0.927355	0.013990804	223
Composite Spike SNAb	Total IgG BA.5 Spike	0.925459	0.013990804	223
Composite Spike SNAb	Total IgG BQ.1.1 Spike	0.959404	0.007799402	223
Composite Spike SNAb	Total IgG Delta Spike	0.950874	0.009619785	223
Composite Spike SNAb	Total IgG JN.1 Spike	0.947865	0.009657231	223
Composite Spike SNAb	Total IgG XBB.1.5 Spike	0.939262	0.013068505	223
Composite Spike SNAb	Total IgG KP.3 Spike	0.913124	0.018750346	223
Composite Spike SNAb	IgG2 WT Spike	0.929881	0.013990804	223
Composite Spike SNAb	IgG3 WT Spike	0.898772	0.021785439	223
Composite Spike SNAb	IgG3 Delta Spike	0.849235	0.045356189	223
Composite Spike SNAb	IgG3 JN.1 Spike	0.841822	0.048661967	223
Composite Spike SNAb	IgG3 KP.3 Spike	0.883463	0.026779157	223
Composite Spike SNAb	IgA1 WT Spike	0.902196	0.021279605	223
Composite Spike SNAb	IgA1 Alpha Spike	0.865011	0.036405921	223
Composite Spike SNAb	IgA1 Beta Spike	0.906236	0.020874697	223
Composite Spike SNAb	IgA1 Delta Spike	0.903722	0.021137256	223
Composite Spike SNAb	IgA1 JN.1 Spike	0.860932	0.038197714	223
Composite Spike SNAb	IgM BA.2 Spike	-0.84498	0.047405558	223
Composite Spike SNAb	IgM BA.5 Spike	-0.90549	0.020874697	223
Composite Spike SNAb	IgM BQ.1.1 Spike	-0.91417	0.018750346	223
Composite Spike SNAb	FcγR2A WT Spike	0.968159	0.007528919	223
Composite Spike SNAb	FcγR2A Alpha Spike	0.949163	0.009657231	223
Composite Spike SNAb	FcγR2A Beta Spike	0.981345	0.005034223	223
Composite Spike SNAb	FcγR2A Delta Spike	0.988566	0.003336056	223
Composite Spike SNAb	FcγR2B WT Spike	0.912051	0.018750346	223
Composite Spike SNAb	FcγR2B Beta Spike	0.962817	0.007528919	223
Composite Spike SNAb	FcγR2B Delta Spike	0.894335	0.022848189	223
Composite Spike SNAb	FcγR3A WT Spike	0.888245	0.024816298	223
Composite Spike SNAb	FcγR3A Alpha Spike	0.93378	0.013068505	223
Composite Spike SNAb	FcγR3A Beta Spike	0.934048	0.013068505	223
Composite Spike SNAb	FcγR3A BA.1 Spike	0.878143	0.029105332	223
Composite Spike SNAb	FcγR3A BQ.1.1 Spike	0.927897	0.013990804	223
Composite Spike SNAb	FcγR3A Delta Spike	0.951398	0.009619785	223
Composite Spike SNAb	FcγR3B WT Spike	0.898853	0.021785439	223
Composite Spike SNAb	FcγR3B Alpha Spike	0.967184	0.007528919	223
Composite Spike SNAb	FcγR3B Beta Spike	0.935955	0.013068505	223
Composite Spike SNAb	FcγR3B BA.1 Spike	0.926229	0.013990804	223
Composite Spike SNAb	FcγR3B BA.2 Spike	0.891647	0.023635954	223
Composite Spike SNAb	FcγR3B BQ.1.1 Spike	0.897421	0.02185973	223
Composite Spike SNAb	FcγR3B Delta Spike	0.979038	0.005034223	223
Composite Spike SNAb	FcγR3B XBB.1.5 Spike	0.937	0.013068505	223

Table S3. Antibody features significantly correlated with SNAb titers from hybrid immunity.

Supplementary Figure 1

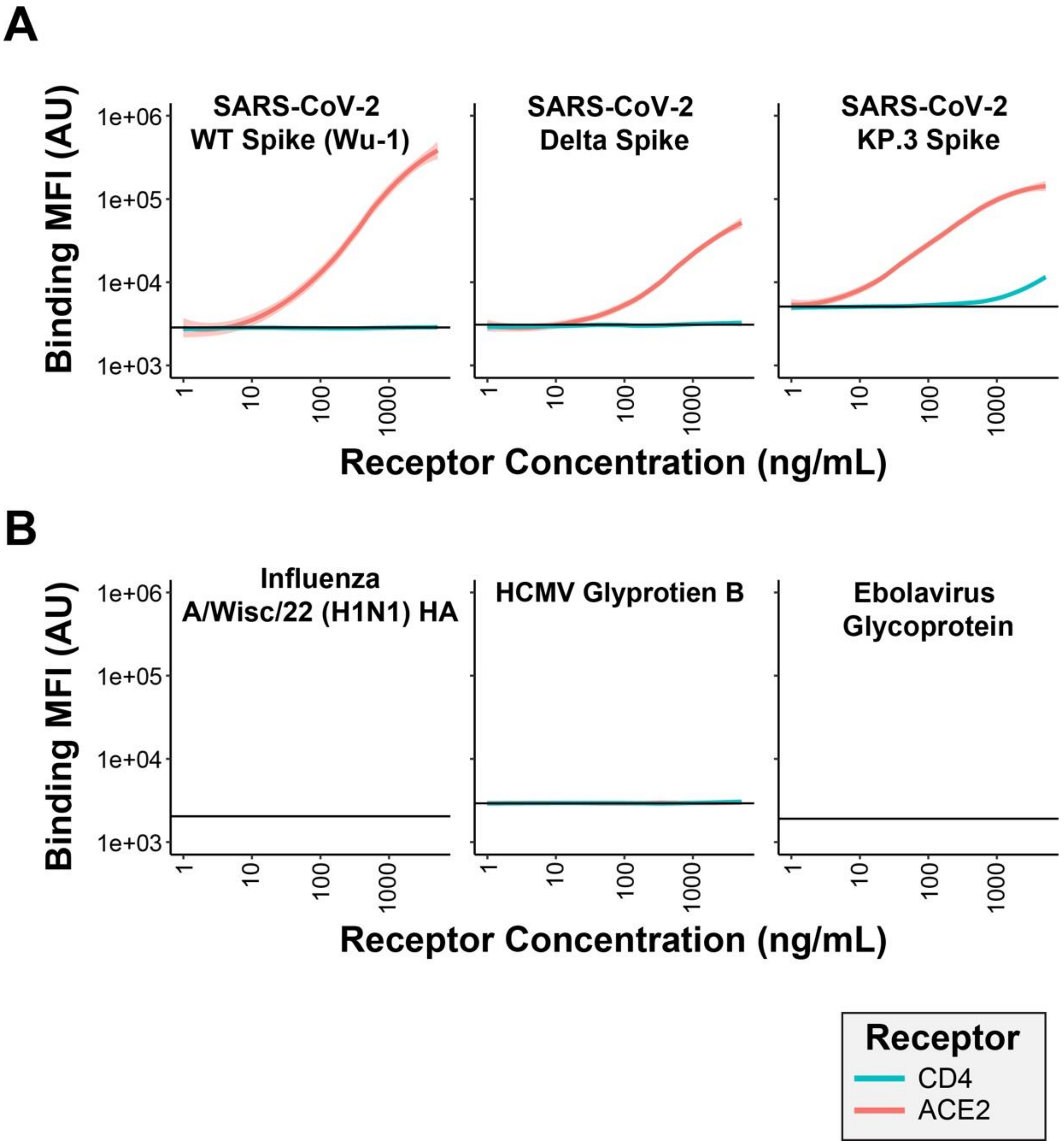

Supplementary Figure 1. Validation of receptor-binding protein specificity and receptor specificity. A) Spike variants were incubated with increasing concentrations of human CD4 (negative binding control) and human ACE2 in a multiplexed manner. Binding levels were quantified through binding MFI in AU. The black line indicates no addition of receptor (lower limit of the blank). B) Binding levels of influenza A/Wisc/22 (H1N1) HA, human cytomegalovirus (HCMV) glycoprotein B, and Ebolavirus glyprotein to CD4 and ACE2. The black line indicates no addition of receptor. All analytes were measured simultaneously.

Supplementary Figure 2

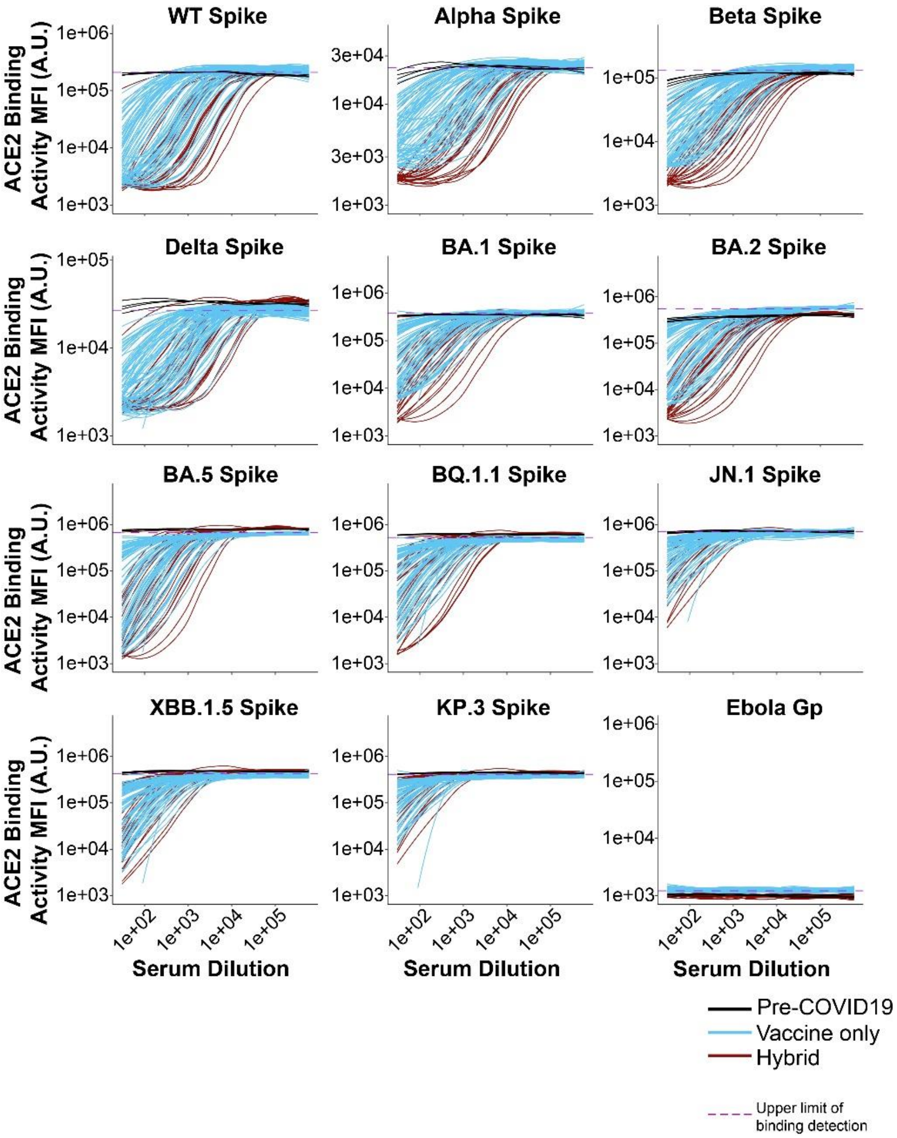

Supplementary Figure 2. Binding levels of Spike variant trimers to ACE2 in the presence of antibody-containing serum samples. Binding of Spike variants to ACE2 was quantified in median fluorescence intensity (MFI) and reported in arbitrary units (A.U.). Absence of detectable binding and completely uninhibited binding models for each variant were made using pre-COVID19 (dark gray), vaccine-immune (blue), and hybrid-immune (red) serum samples. Uninhibited binding was modeled through the upper limit of binding detection for each Spike trimer to ACE2 (dashed purple line). Ebolavirus glycoprotein (Ebola GP) was used as a negative ACE2 binding control. Each colored line represents the Spikes binding to ACE2 in the presence of serum at the indicated dilution (x-axis). Individual samples were done in technical replicates, and mean values for each dilution were used to generate the inhibition curve. The color legend is shown at the bottom. All analytes were measured simultaneously.

Supplementary Figure 3

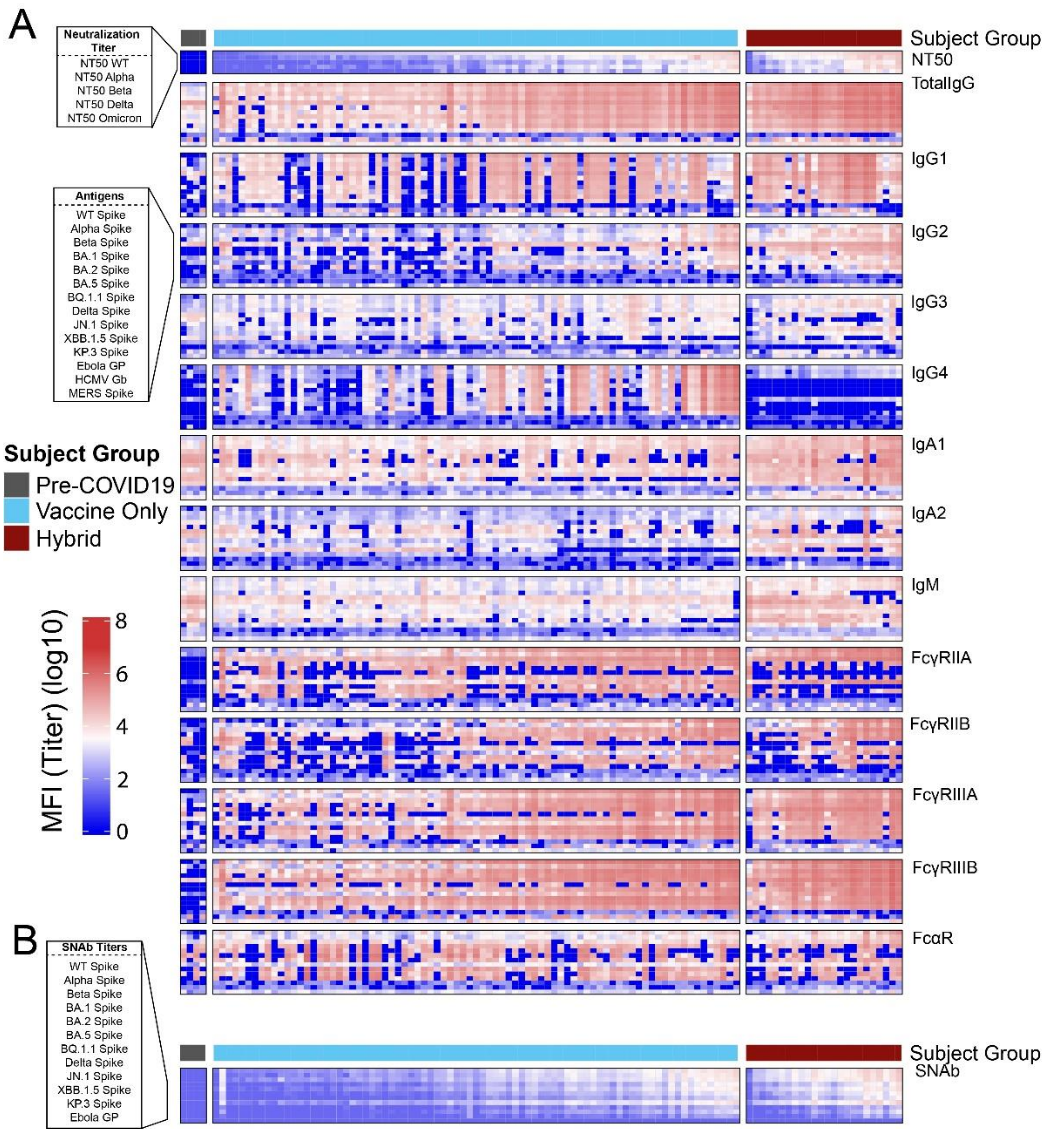

Supplementary Figure 3. Non-normalized systems serology and SNAb levels. A) Antibody binding arrays SARS-CoV-2 Spike variants and control antigens shown on the left. Non-normalized values are shown. Each box represents the mean of technical replicates. All values were log 10 transformed, and a heatmap legend is shown on the left. B) Same as A, but for SNAb values for the targets shown on the left. Binding inhibition is then transformed into a value ranging from 0-100%, which is shown in **Figure 1**.

Supplementary Figure 4

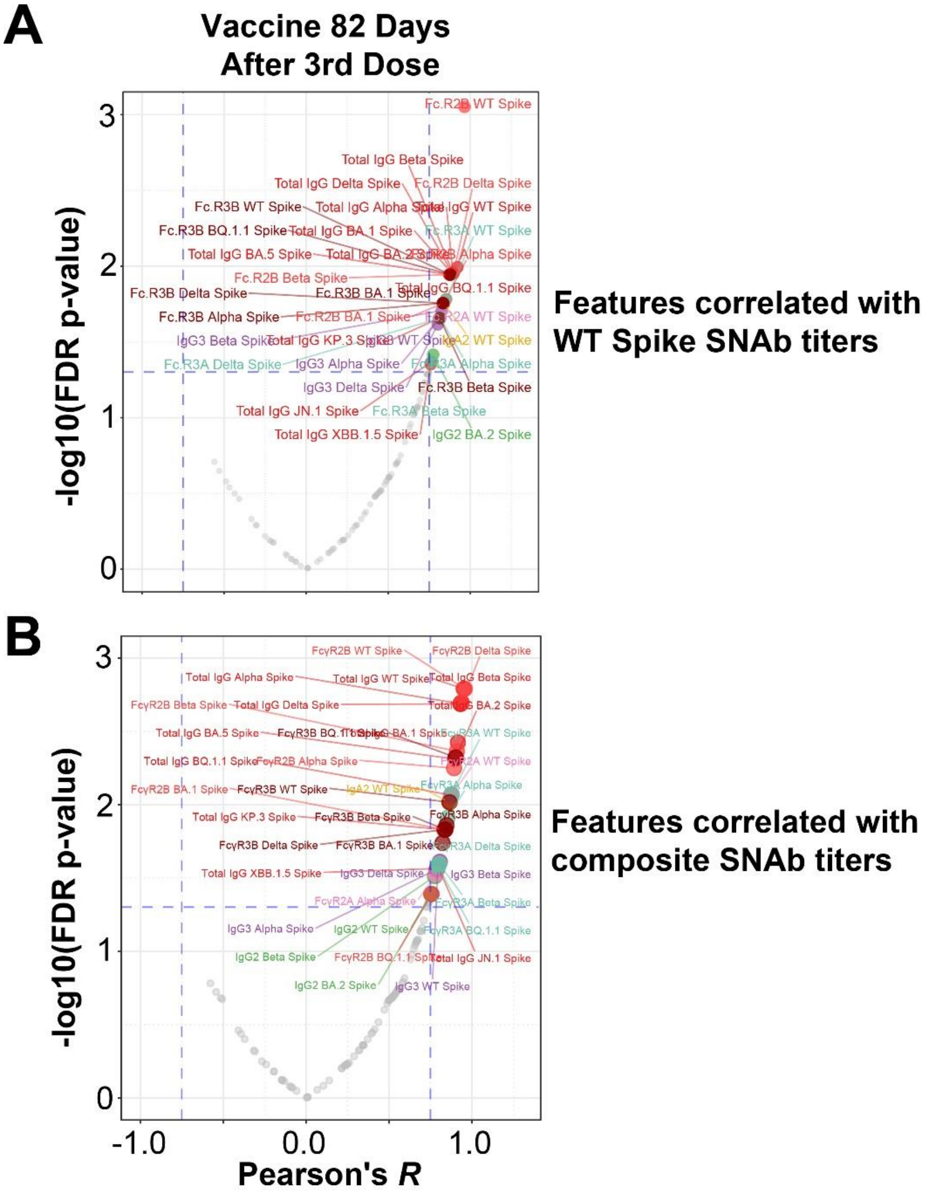

Supplementary Figure 4. Antibody features correlated with vaccine-only immunity SNAb titers. A) Pearson's correlation coefficients (*R*) for all antibody features against WT Spike SNAb titers were plotted using a volcano plot for three dose vaccine recipients 82 days after the 3rd dose. Shown on the x-axis is the *R* value to WT Spike SNAb titers, ranging from -1 to 1. Shown on the y-axis is the FDR adjusted p-value in -log 10. Thresholds for significance were an $|R| \geq 0.75$ and an FDR p-value < 0.05 (dashed blue lines). All features passing these thresholds were highlighted and labeled; all other features were left gray and not labeled. B) Same as a, but for composite SNAb titers (summed titers to all Spike variant trimers) for three dose vaccine recipients 82 days after the 3rd dose.
